# ALDH2/SIRT1 Contributes to Type 1 and Type 2 Diabetes-Induced Retinopathy through Depressing Oxidative Stress

**DOI:** 10.1155/2021/1641717

**Published:** 2021-10-23

**Authors:** Mengshan He, Pan Long, Tao Chen, Kaifeng Li, Dongyu Wei, Yufei Zhang, Wenjun Wang, Yonghe Hu, Yi Ding, Aidong Wen

**Affiliations:** ^1^Department of Pharmacy, Xijing Hospital, Fourth Military Medical University, Xi'an, 710032 Shaanxi, China; ^2^Department of Ophthalmology, The General Hospital of Western Theater Command, Chengdu, 610083 Sichuan, China; ^3^Center of Clinical Aerospace Medicine, Fourth Military Medical University, Xi'an, 710032 Shaanxi, China; ^4^Experiment Teaching Center, Fourth Military Medical University, Xi'an, 710032 Shaanxi, China; ^5^The Air Force Hospital from Northern Theater PLA, Shenyang, 110092 Liaoning, China; ^6^Clinical Medical College & Affiliated Hospital of Chengdu University, Chengdu, 610081 Sichuan, China

## Abstract

Clinical observations found vision-threatening diabetic retinopathy (DR) occurs in both type 1 diabetes mellitus (T1DM) and type 2 diabetes mellitus (T2DM) patients, but T1DM may perform more progressive retinal abnormalities at the same diabetic duration with or without clinical retinopathy. In the present study, T1DM and T2DM patients without manifestations of DR were included in our preliminary clinical retrospective observation study to investigate the differentiated retinal function at the preclinical stage. Then, T1DM and T2DM rat models with 12-week diabetic duration were constructed to explore the potential mechanism of the discrepancy in retinal disorders. Our data demonstrated T1DM patients presented a poor retinal function, a higher allele frequency for ALDH2GA/AA, and a depressed aldehyde dehydrogenase 2 (ALDH2) activity and silent information regulator 1 (SIRT1) level, compared to T2DM individuals. In line with this, higher amplitudes of neurovascular function-related waves of electroretinograms were found in T2DM rats. Furthermore, the retinal outer nuclear layers were reduced in T1DM rats. The levels of retinal oxidative stress biomarkers including total reactive oxygen species, NADPH oxidase 4 and mitochondrial DNA damage, and inflammatory indicators covering inducible/endothelial nitric acid synthase ratio, interleukin-1, and interleukin-6 were obviously elevated. Notably, the level of retinal ALDH2 and SIRT1 in T1DM rats was significantly diminished, while the expression of neovascularization factors was dramatically enhanced compared to T2DM. Together, our data indicated that the ALDH2/SIRT1 deficiency resulted in prominent oxidative stress and was in association with DR progression. Moreover, a differentiating ALDH2/SIRT1 expression may be responsible for the dissimilar severity of DR pathological processes in chronic inflammatory-related T1DM and T2DM.

## 1. Introduction

Diabetes mellitus (DM) has been a severe health-killer, and how to manage itself and its comorbidities is becoming a global concern [1]. It is estimated that the prevalence of DM will rise sharply from the 464 million cases in 2019 to 700 million by 2045 [2]. Diabetic retinopathy (DR) is a common neurovascular complication of DM and has become one of the most frequent causes of blindness in the working-age population worldwide [3, 4].

In clinical, diabetic retinopathy occurs both in type 1 diabetes mellitus (T1DM) featured with severe insulin deficiency and type 2 diabetes mellitus (T2DM) featured with insulin resistance and/or impaired insulin secretion. Astonishingly, individual lifetime risk of DR is up to 90% in T1DM patients and 50–60% in T2DM patients [5–7]. As we have known, the frequency of the progression to DR is influenced by multifactors, such as age, persisted hyperglycemia, and duration of diabetes [8]. Interestingly, the severity of DR denoted by various clinical symptoms existed an obvious difference between T1DM and T2DM at the nearly duration point [9]. However, there are few systematic researches about the comparison of DR features at preclinical or clinical stages caused by T1DM and T2DM, respectively. Here, we would like to study preclinical DR patients who suffered from T1DM or T2DM and established two corresponding animal models to determine the different retinal manifestations. Specifically, via exploring the representative retinal structure, function, and biochemical features of T1DM and T2DM animal models at the same duration, we may reconfirm the clinical finding and further uncover the substantial mechanism of different retinal disorders between T1DM and T2DM.

Intricate interconnecting biochemical pathways are implicated in modulating the pathophysiology of diabetic retinopathy [10]. Among those related mechanisms, oxidative stress (OS), as a consequence of hyperglycemia-provoked overproduction of reactive oxygen species (ROS), is believed as the promising pathway to be responsible for DR progression. As we all know, elevated ROS can result in massive cellular structure damage and mitochondrial dysfunction. Moreover, OS has been postulated as the underlying stressor linking other relative mechanisms which could be related to retinal structural, functional, and metabolic abnormalities in DR process [11, 12]. Specifically, NADPH oxidases (NOX4) could induce OS-correlated mitochondrial impairment and lipid peroxidation (4-hydroxynonenal) in neurodegeneration disease [13].

Recently, studies found that diabetic retinal cells possessed an impaired aldehyde detoxifying capacity, making them especially be prone to aldehyde damages [14, 15]. And elevated lipid aldehydes such as 4-hydroxynonenal (4-HNE) can induce carbonyl stress and mitochondrial damage and accumulate advanced glycation end products (AGEs) [16, 17]. Interestingly, aldehyde dehydrogenase 2 (ALDH2), the mitochondrial isoform of aldehyde dehydrogenases, plays vital roles in clearing acetaldehyde and endogenous lipid aldehydes. Furthermore, epidemiological studies suggest that the gene polymorphism of ALDH2 rs671 in the population is related to DR susceptibility and development [18, 19]. Indeed, our previous study verified that ALDH2 could alleviate early stage aged diabetic retinal damage through upregulating silent information regulator 1 (SIRT1) signaling and depressing oxidative stress reaction [20].

SIRT1 wildly expresses throughout the retinal cells' nuclear, which plays a pivotal role in regulating cell metabolism and mitochondrial homeostasis. Moreover, a body of evidences from our group and others demonstrated that SIRT1 showed strongly protective roles against DR development through attenuating oxidative stress, inflammation, and apoptosis [20, 21]. Additionally, SIRT1 is well-recognized as a redox-sensitive enzyme and tightly affected by cellular oxidative stress conditions [21, 22]. To be specific, the disturbance of oxidative stress, including carbonyl stress (induced by lipid aldehydes), can interfere SIRT1 via downregulating nicotinamide adenine dinucleotide (NAD) levels, decreasing posttranslational modification's ability, and inducing protein-protein interaction disorders [21, 22].

To this regard, ALDH2/SIRT1 may serve as one of the potent molecules to restore the redox homeostasis and mitochondrial health. Lately, our work further revealed that ALDH2 could diminish oxidative stress-related apoptosis and inflammation in naturally aged mouse retinas [23]. In fact, proinflammatory cytokines (interleukin-6), nitric oxide synthase (iNOS), and vascular endothelial growth factor-*α* (VEGF-*α*) directly or indirectly stimulated by oxidative stress can be found in varying ocular diseases including age-related macular degeneration, retinitis pigmentosa, and diabetic retinopathy [24–26]. To be specific, VEGF-*α* is a powerful angiogenic inducer which is not only responsible for ocular neovascularization but also implicated in the increase of macrophage and monocyte chemotaxis [27]. As a result of ROS overabundance and a compromised defense against oxidative stress, aggravated retinal neuron (photoreceptor cells) and capillary disorders can accelerate the development of DR pathogenesis. Accordingly, elucidating the distinction of such typical biomarkers would be conducive to uncover the underlying mechanisms in T1DM/T2DM-related DR and make more targeted therapeutical strategies.

Therefore, the goal of this preliminary clinical retrospective observation study was to determine whether preclinical stage of DR in T1DM and T2DM would exist retinal structural or functional difference. Moreover, rat models, involving single high-dose streptozotocin- (STZ-) induced T1DM and high-calorie diet combined with low-dose STZ-induced T2DM, were applied to investigate the retinal dysfunction characteristics and further explore the potential mechanism in the pathological processes of T1DM and T2DM.

## 2. Materials and Methods

### 2.1. Study Subjects

A total of 85 individuals were included in this preliminary clinical retrospective observation study, including 70 cases with diabetes mellitus and 15 healthy individuals as controls (CON). Diabetic patients were classified into two cohorts by the type of diabetes mellitus, and it included 15 type 1 diabetes mellitus (T1DM) and 55 type 2 diabetes mellitus (T2DM). All of the subjects had no clinical manifestations of DR such as microaneurysms, macular edema, or neovascularization, which was confirmed by fundus fluorescein angiography, slit lamp microscope, and optical coherence tomography (OCT) according to certified ophthalmologists, and the best-corrected visual acuity (BCVA) was beyond 20/20. Additionally, the demographic and clinical characteristics of the study participants such as age, sex, body mass index (BMI), fasting blood glucose, diabetic duration, retina thickness, full-field electroretinograms (ERG) test, and therapies for diabetes were recorded. Subjects with uncontrolled hypertension, acute or chronic systematical inflammatory disease, and maturity-onset diabetes of the young were excluded. The cases were collected from the Department of Ophthalmology in the General Hospital of Western Theater Command through 2018.01-2020.02. All participants gave written informed consent prior to study participation, and this retrospective case-control study was approved by the Ethics Committee of the General Hospital of Western Theater Command for human studies.

### 2.2. Genotyping of ALDH2 Genotypes in Preclinical DR Patients

The blood samples were collected from subjects following a 12-hour fast, and genomic DNA specimens were extracted by using a commercial DNA extraction and purification kit (Shenggong, China) following the manufacturer's protocols. Genotyping was performed on coded genomic DNA samples following a previous study [28]. The ALHD2 rs671, the most well-known functional single nucleotide polymorphism (SNP) acting as a coenzyme binding site [29], was selected as a typical ALDH2 genotyping marker in this study. Individuals carrying wild-type homozygote of the GG (ALDH2∗1/∗1) have a full enzyme activity, whereas participants with mutant heterozygote of the GA (ALDH2∗1/∗2) or mutant homozygote of the AA (ALDH2∗2/∗2) genotype have reduced or little enzyme activity.

### 2.3. The Determination of Serum ALDH2 Activity and SIRT1 Expression in Preclinical DR Patients

The blood serum samples were collected and assayed for ALDH2 activity using a commercial colorimetric kit (#ab115348; Abcam) according to the manufacturer's protocol. The specific method was accordant with previous study [30]. The SIRT1 level was estimated by enzyme-linked immunosorbent assay (ELISA) according to the manufacturer's protocols. The ELISA kit for human SIRT1 was obtained from Abcam (#ab171573). Plate readings were done on a Multiskan® GO Microplate Reader from Thermo Fisher (Basingstoke, UK). Standard solutions in the kit were used to normalize the ELISA values for each plate.

### 2.4. Animals and T1DM and T2DM Rats' Model

Male Sprague Dawley (SD) rats were purchased from the Laboratory Animal Center of Fourth Military Medical University in Xi'an, China (license No. 2014270138S). Rats were housed under standard laboratory conditions, and all experiments were performed in compliance with the ARVO Statement for the Use of Animals in Ophthalmic and Vision Research. Studies were approved by the research ethics committee for the care and use of laboratory animals at the Fourth Military Medical University. 40 SD rats (6-8 weeks old, male, 200-240 g) were fed with a high-fat and high-calorie diet (breeding rodent material 54.6%, lard 16.9%, sucrose 14%, casein 10.2%, premix 2.1%, and maltodextrin 2.2%) for 4 weeks, as previously described [20]. Then, the T2DM rats' model was induced by an intraperitoneal injection of streptozotocin (STZ) (45 mg/kg body weight) (Sigma, USA) after a 4-week specific diet. Simultaneously, to construct the T1DM rats' model with the same diabetic duration, 40 age-matched SD rats (10-12 weeks old, male, 320-360 g) were intraperitoneally injected with a single dose of STZ (60 mg/kg body weight). Seventy-two hours post-STZ injection, blood glucose level was measured by ACCU-CHEK Performa (Roche, Germany) and rats showing a blood glucose level above 16.7 mmol/L were considered as DM and selected for the study. Diabetic rats were divided into two groups: T1DM group (*n* = 30) and T2DM group (*n* = 30), both groups treated with a high-fat and high-calorie diet afterwards. And 30 age-matched SD rats (10-12 weeks old, 320-360 g) served as the CON group.

### 2.5. Full-Field Electroretinograms (ffERG) and Fundus Fluorescein Angiography (FFA) Detection in Diabetic Rats

Full-field electroretinography (ffERG) and fundus fluorescein angiography (FFA) measurements were performed according to the International Society for Clinical Electrophysiology of Vision (ISCEV) guidelines as described earlier [31]. Briefly, dark-adapted (12 h) rats were anesthetized as previously described [20] and scotopic recordings were performed under a dim red-light condition. Electrical responses were recorded with custom-made silver chloride electrodes as previously described [20]. The ERG items, including dark-adaptation 3.0 response and dark-adaptation 3.0 oscillatory potential response, were evaluated. Subsequently, anesthetized rats underwent FFA testing using HRAplusII (Heidelberg, Germany) after intraperitoneal injecting 0.1 mL/100 g 10% fluorescence sodium (Baiyunshan Mingxing Corporation, China). Sodium fluorescein intravenous imaging time was obtained to evaluate retinal vessel function.

### 2.6. HE Staining Detection in Diabetic Rats

Retinal histological alterations were visualized by HE staining of rat eye sections. Rats were euthanized by a lethal dose of sodium pentobarbital, and the eyeballs were rapidly removed and enucleated. The eyes were kept immersed for at least 48 h at 4°C in a fixative solution containing 4% paraformaldehyde and embedded in paraffin blocks. 4 *μ*m sections were prepared in the standard manner and stained with HE staining. The layers of outer nuclear layer (ONL) and inner nuclear layer (INL) cells and the thickness of inner and outer segment layer (IS/OS) were calculated for morphometric analysis. Color micrographs were photographed under a digital imaging system.

### 2.7. Detection of Apoptosis in Diabetic Rats

To determine apoptosis in the retina, we performed TdT-mediated dUTP nick-end labeling (TUNEL) assay labeling the nuclear cut ends of DNA fragments in apoptotic (or necrotic) cells. The detection was conducted following the manufacturer's instructions. DAPI (100 ng/mL) was added in the fluorescent staining procedure to label the nuclei of retinal cells. The fluorescence intensity of the apoptotic signal was quantified by ImageJ software.

### 2.8. Real Time-Quantitative PCR (RT-qPCR) Analysis in Diabetic Rats

Total RNA was isolated from the retinal samples collected at 12-week diabetic duration, using TRIzol reagent. RNA was converted to cDNA by synthesis according to the manufacturer's protocol of the RevertAid M-MuLV cDNA synthesis kit (Thermo Scientific™, EP0733, USA). Real-time polymerase chain reaction (RT-PCR) was performed on the ABI StepOnePlus Real-Time PCR device with FastStart Universal SYBR-Green Master kit (Roche, Germany). GAPDH was used as a housekeeping gene. Primers were as follows: 5′-GAGCAACGTCACTATGCAGATC-3′ (forward) and 5′-TTTCTCCGCTCTGAACAAGG-3′ (reverse) for VEGF-*α*, 5′-TGGATTTGGACATGGTTCTGA-3′ (forward) and 5′-GCGGGTGTAGCTGAAGAAGT-3′ (reverse) for ALDH2, 5′-GCTCGCCTTGCTGTGGACTTC-3′ (forward) and 5′-GTGACACAGAGATGGCTGGAACTG-3′ (reverse) for SIRT1, and 5′-CAAGTTCAACGGCACAGTCAA-3′ (forward) and 5′-CGCCAGTAGACTCCACGACA-3′ (reverse) for GAPDH. Quantification of VEGF-*α*, ALDH2, and SIRT1 mRNA expressions was calculated by normalizing with the GAPDH mRNA expression. Analysis was performed using the 2^−*ΔΔ*Ct^ method.

### 2.9. Immunofluorescence Staining in Diabetic Rats

The immunofluorescence staining and quantitation of VEGF-*α*, ALDH2, SIRT1, SOD1, eNOS, iNOS, and VEGF Receptor2 in rat retinas were performed as described in our previous study [20]. In brief, after deparaffinization and antigen restoration, eye sections were blocked in 1% bovine serum albumin for 1 h and stained overnight at 4°C with VEGF-*α* (1 : 200; #GTX102643, GeneTex), ALDH2 (1 : 200; #ab108306, Abcam,), SIRT1 (1 : 200; #ab110304, Abcam), NOX4 (1 : 200; #ab133303, Abcam), SOD1 (1 : 200; #ab51254, Abcam), eNOS (1 : 200; #ab5589, Abcam), iNOS (1 : 200; #18985-1-AP, Proteintech Group, Inc.), and VEGF Receptor2 (1 : 200; #9698, Cell Signaling Technology) primary antibody. Then, the slides were incubated with HRP-IgG (H + L) secondary antibody (#EK022, #EK012, Zhuangzhibio, China) at 1 : 200 dilution for 1 hour, and the nuclei were stained with 4-6-diamidino-2-phenylindole (DAPI). Images were captured by a fluorescence microscope.

### 2.10. Immunoblotting in Diabetic Rats

The whole retina was dissected, and protein was isolated as described previously [23]. Sodium dodecyl sulfate-polyacrylamide gel electrophoresis (SDS-PAGE) of retinal proteins was performed with Tris-glycine-SDS running buffer (NCM Biotech, China). Next, the proteins from the gels were transferred onto the PVDF membrane (Millipore, US) with electroblotting. After blocking with 5% nonfat milk solution, membranes were incubated with ALDH2 (1 : 1000; #ab108306, Abcam), VEGF-*α* (1 : 1000; #ab46154, Abcam), SIRT1 (1 : 1000; #ab110304, Abcam), Bax (1 : 1000; #ab32503, Abcam), Bcl-2 (1 : 1000; #ab182858, Abcam), Caspase 3 (1 : 1000; #19677-1-AP, Proteintech Group, Inc.), 4-HNE (1 : 1000; #ab46545, Abcam), hypoxia-inducible factor-1*α* (HIF-1*α*) (1 : 1000; #ab1, Abcam), VEGF Receptor2 (1 : 1000; #9698, Cell Signaling Technology), and GAPDH (1 : 1000; #5174, Cell Signaling Technology) overnight at 4°C. Binding of HRP-conjugated secondary antibody (1 : 10000; #EK020, #EK010; Zhuangzhibio, China) was observed using enhanced chemiluminescence (Thermo Fisher Scientific). All blots were quantified by densitometry using ImageJ software (NIH).

### 2.11. Determination of the Retinal Total ROS and Mitochondrial DNA Damage in Diabetic Rats

The fluorescent probe 2′,7′-dichlorofluorescein diacetate (DCHFDA; Sigma, USA) was applied to quantify total ROS levels of the retina. Specifically, 10 *μ*g protein extracted from the retina was incubated with 2 *μ*mol/L DCHFDA for 10 minutes and the resultant fluorescence intensity was detected at excitation wavelength of 485 nm and emission wavelength of 530 nm [32, 33]. Mitochondria were isolated from retina tissues by using the Mitochondria Isolation kit (Sigma, USA) in compliance with the manufacturer's protocols. Total DNA was isolated with a DNeasy blood and tissue kit (Qiagen, USA), and extended-length PCR was performed by amplification of long and short regions of mitochondrial DNA (mtDNA). Un-Scan-It Gel digitizing software was used to capture the intensity of PCR gel photographs, and then, the ratio of the long (13.4 kb) to short fragment (210 bp) of PCR amplicons was estimated. The primers of mtDNA long were 5′-AAAATCCCGCAAACAATGACCACCCC-3′ (forward) and 5′-GGCAATTAAGAGTGGGATGGAGCCAA-3′ (reverse). The primers of mtDNA short were 5′-CCTCCCATTCATTATCGCCGCCCTTGC-3′ (forward) and 5′-GTCTGGGTCTCCTAGTAGGTCTGGGAA-3′ (reverse). The mtDNA damage was denoted by the decreased ratio of long to short amplicons [33].

### 2.12. Determination of Inflammatory Parameters in Diabetic Rats

Retinal tissues were collected at 12 weeks post diabetic rat model established. Interleukin-1 (IL-1) and interleukin-6 (IL-6) levels in retinas and insulin in serum were assessed using a commercially available enzyme-linked immunosorbent assay (ELISA) kit from Westang Bio-Tech Co., LTD. (Shanghai, China) according to the manufacturer's instructions.

### 2.13. Statistical Analysis

The statistical software SPSS, version 19.0 (IBM Co., USA), was applied to perform the statistical analysis. Student's *t*-test was used for two-group comparison, and one-way ANOVA followed by Fisher's Least Significant Difference (LSD) post hoc analysis was performed to examine the statistical differences among multiple groups. The chi-squared test (Fisher's exact test) was used to analyze the distributions of allele and genotype frequencies. Categorical variables were expressed as percentages, and continuous data are presented as mean ± standard deviation (SD). All *p* values ≤ 0.05 were considered as statistically significant.

## 3. Results

### 3.1. The Characteristics of Preclinical DR Patients

The general characteristics of diabetic patients with no clinical manifestation of DR are presented in Table 1. The diabetic patients were classified into two cohorts by type of diabetes, and there were 15 patients with T1DM and 55 patients with T2DM. Moreover, 15 healthy individuals were included as the control (CON). The blood glucose levels exhibited no statistically significant difference (fasting blood glucose level < 10 mmol/L) between T1DM and T2DM patients, because of a well-maintained blood glucose control by insulin therapy and/or oral hypoglycemic drugs. Besides, whole retinal thickness showed no statistically significant difference among T1DM, T2DM, and CON individuals. Noteworthily, the T1DM patients in DR preclinical period had a significantly lower ERG amplitude, especially in b wave (dark-adapted 3.0 response) and OPs2 wave, when compared to the T2DM and CON individuals (all *p* < 0.05). However, the b wave (dark-adapted 3.0 response) of the ERG test in T2DM and CON individuals showed no significant difference (*p* > 0.05).

### 3.2. The Gene Polymorphisms of ALDH2 in Preclinical DR Patients

The above results indicated that the retinal function had been decreased more in T1DM compared with T2DM before clinically detectable DR. To investigate the potential role of ALDH2, ALDH2 gene types (ALDH2 rs671 polymorphisms: including ALDH2GG, ALDH2GA, and ALDH2AA) in different cohorts were detected. It was known that those who carried an A allele are related to deficient ALDH2 activity. As shown in Table 2, we found the number of patients or healthy individuals with ALDH2GA/AA genotype was small, and there is no significant difference in the distribution of ALDH2 genotypes. However, the allele frequencies for ALDH2GA/AA in T1DM patients were statistically more than T2DM patients and CON individuals (all *p* < 0.05). In contrast, there was no significant difference in ALDH2GA/AA genotype distribution between T2DM patients and healthy individuals (*p* > 0.05). Taken together, our data suggested that the risk of DR development in patients with T1DM was potentially associated with ALDH2 deficiency.

### 3.3. ALDH2 Activity and SIRT1 Expression in Preclinical DR Patients

**Figure 1:**
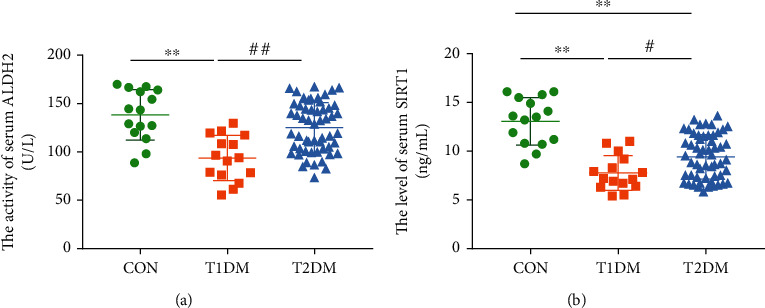
ALDH2 activity and SIRT1 expression in preclinical DR patients and healthy individuals; (a) the activity of serum ALDH2; (b) the level of serum SIRT1. Values are presented as mean ± SD, *n* = 15, 55, and 15. ^∗∗^*p* < 0.01: T1DM cohort and T2DM cohort vs. CON cohort; ^#^*p* < 0.05 and ^##^*p* < 0.01: T2DM cohort vs. T1DM cohort.

To further explore whether the ALDH2 activity could be related to preclinical DR patients' retinal function, serum ALDH2 activity was estimated. As showed in Figure 1(a), T1DM patients presented a significantly lower level of serum ALDH2 activity compared with CON individuals (*p* < 0.01). Interestingly, the serum ALDH2 activity in T1DM patients was evidently decreased compared with that in T2DM patients (*p* < 0.01). Additionally, the potential associations between ALDH2 and SIRT1 were once revealed by our previous work [20]. As shown in Figure 1(b), the serum SIRT1 level in T1DM patients was downregulated compared to T2DM patients and CON individuals (all *p* < 0.05). Moreover, there existed a significant difference in serum SIRT1 level between T2DM patients and CON individuals (*p* < 0.05).

### 3.4. The Blood Glucose Level, Insulin Concentration, and Retinal Function in Diabetic Rats

**Figure 2:**
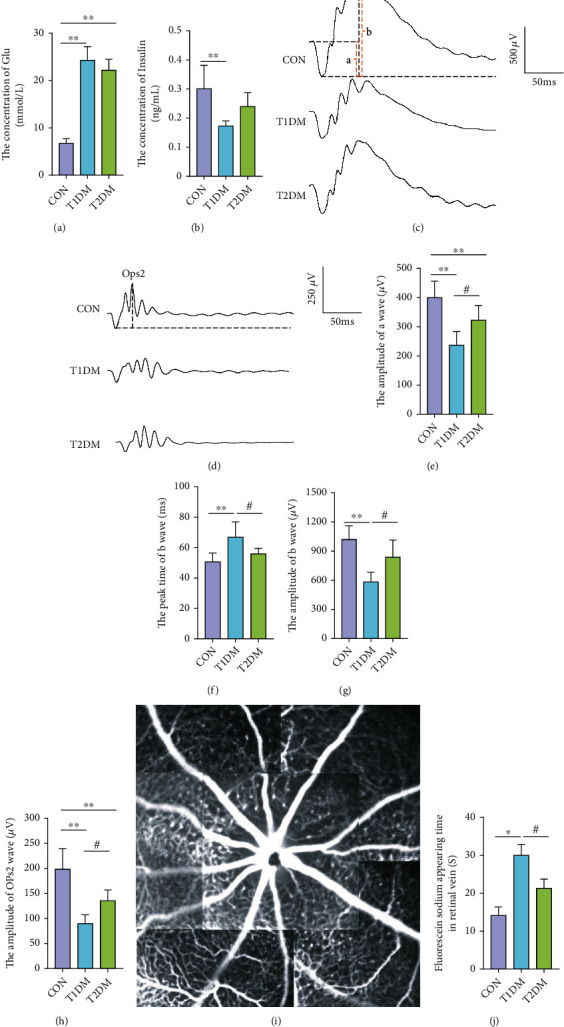
The retinal electrical activity and retinal vessel microcirculation function in the CON, T1DM, and T2DM groups after a 12-week study: (a) the concentration of blood glucose; (b) the concentration of blood insulin; (c) presentive images of a, b wave (dark-adaptation 3.0 response) and (d) OPs2 wave (dark-adaptation 3.0 oscillatory potential response); (e) the amplitude of a wave; (f) the peak time of b wave; (g) the amplitude of b wave; (h) the amplitude of OPs2 wave; (i) the representative fundus fluorescein angiography picture; (j) the fluorescein sodium appearing time in the retinal vein. Values are presented as mean ± SD, *n* = 15 or 6, respectively. ^∗^*p* < 0.05 and ^∗∗^*p* < 0.01: the T1DM group and the T2DM group vs. the CON group; ^#^*p* < 0.05: the T2DM group vs. the T1DM group.

The blood glucose of the CON group was 6.24 ± 0.97 mmol/L at the initial. And the blood glucose level of the T1DM group was 19.67 ± 3.85 mmol/L, and the T2DM group was 20.23 ± 4.26 mmol/L. There existed no significant difference between the T1DM group and the T2DM group (*p* > 0.05). After a 12-week study, the level of blood glucose in the T1DM group was 23.84 ± 2.85 mmol/L and the T2DM group was 24.51 ± 4.63 mmol/L with no statistically significant difference (*p* > 0.05) (Figure 2(a)). Moreover, the level of blood insulin in the T1DM group was 0.17 ± 0.02 ng/mL and that in the T2DM group was 0.24 ± 0.05 ng/mL, while the blood insulin level in the CON group was 0.30 ± 0.08 ng/mL after a 12-week study (Figure 2(b)). As we could see, the blood insulin level in the CON and T2DM groups was all higher than the T1DM group (all *p* < 0.05), while there existed no significant difference between the CON group and the T2DM group (*p* > 0.05).

To evaluate the retinal function of both types of DM at 12 week's durations, ffERG and FFA were performed. As shown in Figures 2(c)–2(e) and 2(h), the amplitude of a wave (dark-adaptation 3.0 response) and OPs2 wave (dark-adaptation 3.0 oscillatory potential response) in both the T1DM and T2DM groups obviously declined at 12-week diabetic duration compared to the CON group (all *p* < 0.05). And the T2DM group showed much better performances in the amplitude of a and OPs2 waves relative to T1DM. Furthermore, Figures 2(f) and 2(g) show the amplitude of b wave (dark-adaptation 3.0 response) in the T1DM group was significantly lower than that in the CON group (*p* < 0.01) and the peak time of b wave (dark-adaptation 3.0 response) in the T1DM group was significantly longer than the CON group (*p* < 0.01). Interestingly, in the T2DM group, the amplitude of b wave (dark-adaptation 3.0 response) was obviously higher than that in the T1DM group (*p* < 0.05); meanwhile, the peak time of b wave was significantly shorter (*p* < 0.05). However, the amplitude and peak time of b wave (dark-adaptation 3.0 response) existed no statistically significant difference between the T2DM group and the CON group (all *p* > 0.05). As for rats' retinal vessel microcirculation function, it is shown in Figure 2(i) and 2(j) that the fluorescein sodium appearing time in the retinal vein in the T1DM group (28.44 ± 4.85 s) was evidently prolonged compared to the CON (14.45 ± 2.24 s) and T2DM groups (18.67 ± 3.28 s) (all *p* < 0.05), whereas the difference between the T2DM group and the CON group did not achieve statistical significance (*p* > 0.05).

### 3.5. Retinal Morphological Structure and Apoptosis in Diabetic Rats

**Figure 3:**
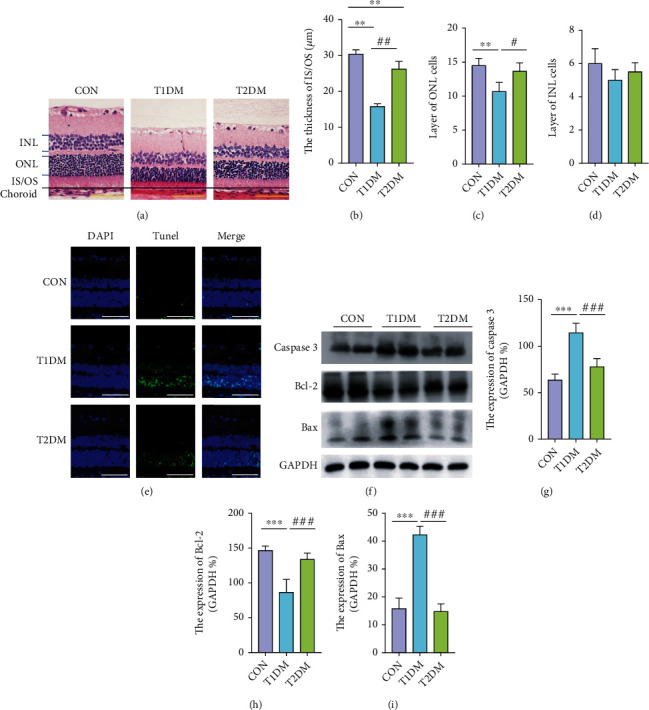
The number of retinal cell layers in the CON, T1DM, and T2DM groups detected by HE staining after a 12-week study: (a) the representative HE staining picture; (b) the thickness of OS/IS layer; (c) the layer of ONL cells; (d) the layer of INL cells; (e) the presented TUNEL images; (f) the representative Western blotting picture of Caspase 3, Bcl-2, and Bax; (g) the relative protein expression of Caspase 3; (h) the relative protein expression of Bcl-2; (i) the relative protein expression of Bax. INL: inner nuclear layer; ONL: outer nuclear layer; IS/OS: inner and outer segment layer; RPE: retinal pigment epithelial. Scale bar: 100 *μ*m. Values are presented as mean ± SD, *n* = 4-6. ^∗∗^*p* < 0.01, and ^∗∗∗^*p* < 0.001: the T1DM group and the T2DM group vs. the CON group; ^#^*p* < 0.05, ^##^*p* < 0.01, and ^###^*p* < 0.001: the T2DM group vs. the T1DM group.

HE staining and TUNEL assay were applied to evaluate retinal morphometric structure changes and determine whether cell apoptosis partly caused this phenomenon. As shown in Figures 3(a) and 3(b), compared with the CON group, an extremely decreasing thickness of photoreceptors' inner and outer segment layer (IS/OS) was observed in both DM groups (all *p* < 0.05). Moreover, T1DM had significantly lesser cell layers of outer nuclear layer (ONL) which consists of photoreceptors' (cone and rod cells) nucleus, compared to the CON group (*p* < 0.05) (Figure 3(c)). Significantly, T1DM exhibited thinner IS/OS and ONL layers relative to T2DM retinas (all *p* < 0.05). When it came to the cell layers of the inner nuclear layer (INL), despite the T1DM group showed a decreasing tendency compared with the CON group, there existed no statistically significant difference among CON, T1DM, and T2DM (*p* > 0.05) (Figure 3(d)). Subsequently, it is shown in Figure 3(e) that apoptosis obviously occurred in T1DM retinas rather than T2DM retinas, especially in photoreceptor layers (ONL), compared with CON retinas according to TUNEL assay (all *p* < 0.01). This was further proved by the prominent elevated expression of proapoptotic molecules Bax and Caspase 3 and depressed antiapoptotic Bcl-2 in T1DM retinas, when compared to the CON and T2DM groups (*p* < 0.05) (Figures 3(f)–3(i)).

### 3.6. Retinal ALDH2 and SIRT1 Expression in Diabetic Rats

**Figure 4:**
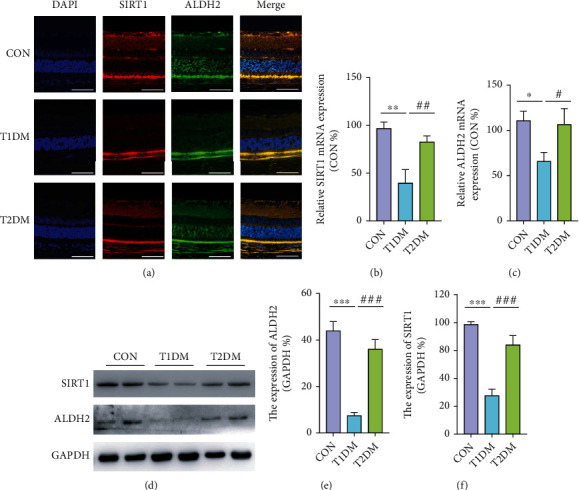
The protein and mRNA expressions of retinal SIRT1 and ALDH2 detected by immunofluorescence, RT-PCR, and Western blotting in the CON, T1DM, and T2DM groups after a 12-week study: (a) the representative immunofluorescence picture of ALDH2 and SIRT1; (b) the relative mRNA expression of ALDH2; (c) the relative mRNA expression of SIRT1; (d) the representative Western blotting picture of ALDH2 and SIRT1; (e) the relative protein expression of ALDH2; (f) the relative protein expression of SIRT1. Scale bar: 100 *μ*m. Values are presented as mean ± SD, *n* = 3-4. ^∗^*p* < 0.05, ^∗∗^*p* < 0.01, and ^∗∗∗^*p* < 0.001: the T1DM group and the T2DM group vs. the CON group; ^#^*p* < 0.05, ^##^*p* < 0.01, and ^###^*p* < 0.001: the T2DM group vs. the T1DM group.

The mRNA expression of retinal ALDH2 and SIRT1 was detected by the RT-PCR method. Meanwhile, the protein expression of ALDH2 and SIRT1 was measured both via immunofluorescence and Western blotting assays. As shown in Figure 4(a), SIRT1 was strongly expressed in ONL, INL, and ganglion cell layer (GCL), which consisted with ALDH2, which was wildly expressed in metabolically active position. The mRNA expression of retinal ALDH2 and SIRT1 in the T1DM group was lesser than the CON and T2DM groups (all *p* < 0.05) (Figures 4(b) and 4(c)). However, the mRNA expression of ALDH2 and SIRT1 in the CON group and the T2DM group existed no significant difference (all *p* > 0.05). Moreover, the protein expression of ALDH2 and SIRT1 in the T1DM group was significantly decreased compared with both the CON and T2DM groups indicated by immunofluorescence and Western blotting detections (all *p* < 0.05) (Figures 4(a) and 4(d)–4(f)). Interestingly, the protein expression of ALDH2 and SIRT1 showed no significant difference between the T2DM and CON groups according to Western blotting (all *p* > 0.05) (Figures 4(d)–4(f)). The outcomes of Western blotting were in accordance with immunofluorescence staining.

### 3.7. Retinal VEGF-*α*, HIF-1*α*, and VEGFR2 Expressions in Diabetic Rats

**Figure 5:**
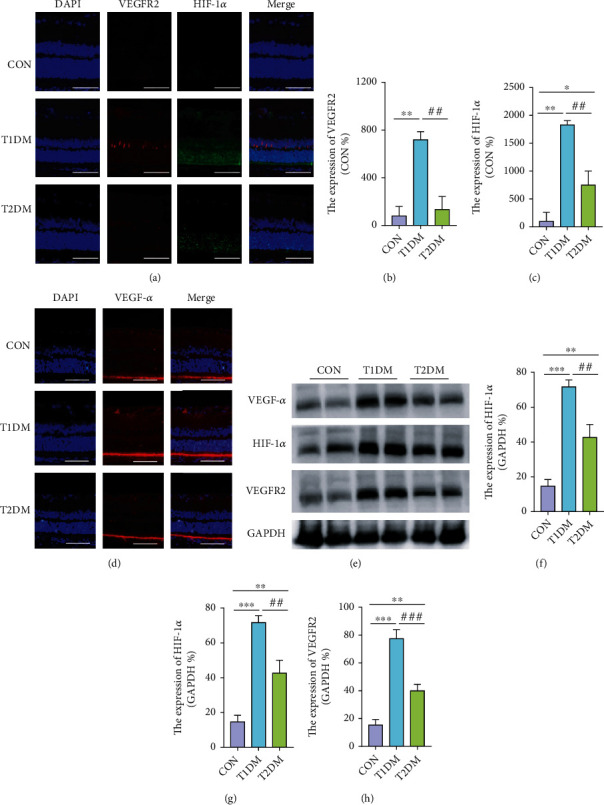
The expressions of retinal VEGF-*α*, HIF-1*α*, and VEGFR2 in the CON, T1DM, and T2DM groups after a 12-week study: (a, d) the representative immunofluorescence picture of VEGF-*α*, HIF-1*α*, and VEGFR2; (b) the relative expression of VEGFR2; (c) the relative expression of HIF-1*α*; (e) the representative Western blotting picture of VEGF-*α*, HIF-1*α*, and VEGFR2; (f–h) the relative protein expression of VEGF-*α*, HIF-1*α*, and VEGFR2. Scale bar: 100 *μ*m. Values are presented as mean ± SD, *n* = 3-4. ^∗^*p* < 0.05, ^∗∗^*p* < 0.01, and ^∗∗∗^*p* < 0.001: the T1DM group and the T2DM group vs. the CON group; ^#^*p* < 0.05, ^##^*p* < 0.01, and ^###^*p* < 0.001: the T2DM group vs. the T1DM group.

To estimate the reaction of neovascularization in the retina, angiogenesis mediators VEGF-*α*, HIF-1*α*, and VEGFR2 were measured at 12-week diabetic duration. As shown in Figures 5(a) and 5(d), VEGF-*α* was strongly expressed in the external limiting membrane (ELM), inner plexiform layer (IPL), and ganglion cell layer (GCL); meanwhile, HIF-1*α* and VEGFR2 immunoreactivities were observed in ONL and INL, and GCL. It was found that the protein expression of VEGF-*α* in T1DM retinas was more than those in the CON and T2DM groups (all *p* < 0.05), while there existed no significant difference between CON and T2DM (all *p* > 0.05) (Figures 5(e) and 5(f)). Moreover, VEGFR2, the angiogenic receptor of VEGF, was extremely increased in T1DM, compared to CON and T2DM (all *p* < 0.05) (Figures 5(b), 5(e), and 5(h)). Additionally, although retinal HIF-1*α*, a VEGF transcriptional regulator, was increased in both types of DM relative to CON as demonstrated in Figure 5(c), T1DM exhibited a much greater increase of HIF-1*α* expression compared to T2DM, which was confirmed by both Western blotting and immunofluorescence (Figures 5(e) and 5(g)). It could indicate that the changes of retinal HIF-1*α* and VEGFR2 levels in T1DM were closely correlated to the elevating expression of VEGF-*α*.

### 3.8. The Retinal iNOS/eNOS Ratio and Inflammation in Diabetic Rats

**Figure 6:**
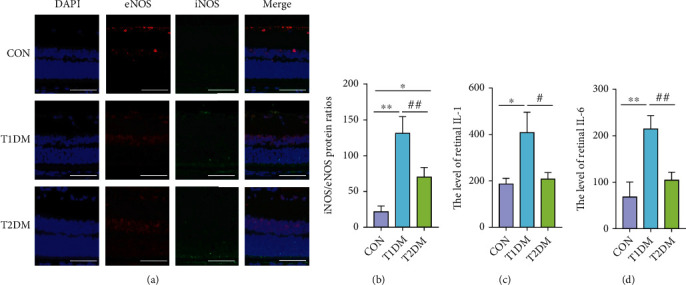
The level of retinal iNOS, eNOS, IL-1, and IL-6 in the CON, T1DM, and T2DM groups after a 12-week study: (a) the representative immunofluorescence picture of iNOS and eNOS; (b) the ratio of iNOS expression to eNOS expression; (c) the level of retinal IL-6; (d) the concentration of retinal IL-1. Scale bar: 100 *μ*m. Values are presented as mean ± SD, *n* = 3-4. ^∗^*p* < 0.05 and ^∗∗^*p* < 0.01: the T1DM group and the T2DM group vs. the CON group; ^#^*p* < 0.05 and ^##^*p* < 0.01: the T2DM group vs. the T1DM group.

Immunofluorescence assay was further performed to identify iNOS/eNOS imbalance in the diabetic retina. According to our study (data not shown), the retinal iNOS level was notably elevated in T1DM (*p* < 0.05) at 12-week diabetic duration, compared to CON and T2DM, whereas there existed no statistically significant difference of retinal eNOS expression among all groups (*p* > 0.05). Surprisingly, the iNOS/eNOS ratio in T1DM and T2DM retinas exhibited an obvious increase compared with CON retinas, which denoted an imbalance of iNOS/eNOS in diabetic retinas, especially in T1DM when compared to T2DM (all *p* < 0.05) ((Figures 6(a) and 6(b)). Undoubtedly, enhanced iNOS level could be a biomarker of the vicious inflammatory milieu in the diabetic retina. Accordingly, proinflammatory cytokines IL-1 and IL-6 were also measured via ELISA assay. As shown in Figure 6(c), retinal IL-1 production in the T1DM group was significantly increased compared with both the CON and T2DM groups (all *p* < 0.01), while there existed no significant difference between the CON group and the T2DM group (*p* > 0.05). As for IL-6, the retinal production in T1DM was also increased compared with both the CON and T2DM groups (all *p* < 0.05), while there existed no significant difference between the CON and the T2DM (*p* > 0.05) at 12-week diabetic duration (Figure 6(d)).

### 3.9. Retinal Oxidative and Mitochondrial DNA Damage Level in Diabetic Rats

**Figure 7:**
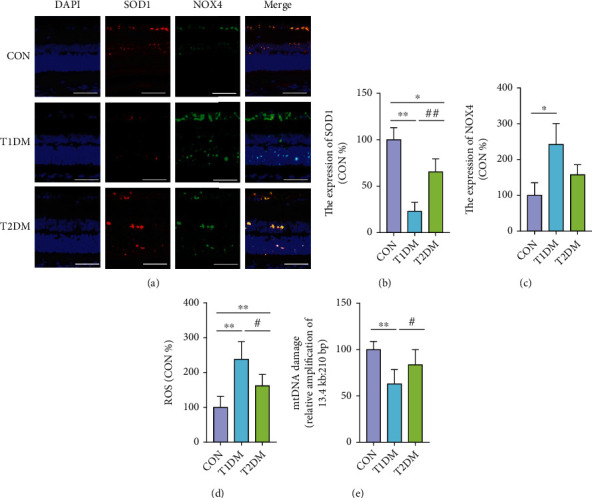
The levels of retinal SOD1, NOX4, total ROS, and mtDNA damage in the CON, T1DM, and T2DM groups after a 12-week study: (a) the representative immunofluorescence picture of SOD1 and NOX4; (b) SOD1 immunofluorescence levels normalized to CON; (c) NOX4 immunofluorescence levels normalized to CON; (d) the retinal total ROS level; (e) damage of mtDNA in the retina. Scale bar: 100 *μ*m. Values are presented as mean ± SD, *n* = 3-4. ^∗^*p* < 0.05 and ^∗∗^*p* < 0.01: the T1DM group and the T2DM group vs. the CON group; ^#^*p* < 0.05 and ^##^*p* < 0.01: the T2DM group vs. the T1DM group.

It was found that antioxidative enzyme superoxide dismutase 1 (SOD1) and ROS-producer NADPH oxidase 4 (NOX4) were both actively expressed mainly in retinal ONL, INL, and GCL (Figure 7(a)). SOD1 was notably declined in T1DM compared to the CON and T2DM retina, while NOX4 was notably elevated in T1DM compared to the CON retina (all *p* < 0.05) (Figures 7(b) and 7(c)). Although there were prominent reductions of retinal SOD1 in T2DM, there existed no statistically significant difference in NOX4 expressions between CON and T2DM. According to Figure 7(d), the retinal total ROS level was notably elevated in both T1DM and T2DM rats at 12-week diabetic duration, while there was a relatively lower level of ROS in the T2DM retina compared to the T1DM retina (all *p* < 0.05). It is known that mtDNA is highly susceptible to oxidative stress. As shown in Figure 7(e), despite increased ROS in the retina, mtDNA was not statistically significantly damaged in the T2DM retina (*p* > 0.05). Nevertheless, evident mtDNA damage was observed in the T1DM retina compared to that in the T2DM and CON groups (*p* < 0.05).

## 4. Discussion

Our clinical investigation found an identifiable retinal dysfunction in both T1DM and T2DM patients at preclinical diabetic retinopathy stage. To our surprise, although the disease duration of T1DM patients was shorter, the retinal function of the patients was seriously poor. Additionally, serum ALDH2 activity and SIRT1 expression in T1DM patients were significantly lower than those in T2DM patients. To further verify this phenomenon, we constructed the T1DM and T2DM rats' model to study the potential difference in retinal morphology, function, and molecular biological marker and explore the possible mechanism.

As we all know, diabetic retinopathy can both trigger retinal microvessel and neuron cell damages. The oscillatory potential (OPs) wave, a component of the electroretinographic test, is regarded as a valuable indicator to evaluate retinal vessel-related disorders (retinal ischemia caused by reduced circulation) [34, 35]. Our results showed retinal vessel microcirculation function in both T1DM and T2DM rats attenuated to a lower level, but T1DM deteriorated much more rapidly and seriously, which has also been proved by increased fluorescein sodium appearing time. In fact, this was in line with other findings which revealed that diminished OPs wave amplitudes were found in persons with DM and no photographic evidence of background retinopathy [35].

In the study, we demonstrated that T1DM and T2DM jeopardized rats' retinas mainly at outer layers. This finding was supported by our several observations: (1) typical thinning occurred at the outer layers/photoreceptor layer (including ONL and IS/OS); (2) the prominent apoptotic cells were largely concentrated at the outer layers; (3) the negative wave of ERG (dark-adaptation 3.0 response) called a wave, reflecting the light absorption activity of the photoreceptors, depressed significantly. So, why was the outer retina extremely vulnerable to hyperglycemia? Primarily, there exists vigorous energy metabolism activity in the outer retina accompanied with a great number of metabolic wastes and mitochondrial damages all the time. The similar finding was also verified, in which, mtDNA damage was obviously occurred and even continued when hyperglycemia state continued in T1DM Wistar rat [36, 37]. Secondly, photoreceptors are fragile in some way, with limited nutritional supply and restricted resistant ability to adverse environments. Finally, the protective threshold of cellular stress response is limited, and the sophisticated cellular metabolism pathways are involved, which results in infaust resistance ability. Interestingly, the inner nuclear layers did not significantly decrease in both T1DM and T2DM rats, and we supposed that despite the obvious dysfunction in photoreceptors, Müller, and bipolar cells, the morphological changes of inner retinal would probably intact at this early stage of DR.

It is widely realized that increasing reactive oxygen species (ROS) generation could impair antioxidant defenses, which decreases the ability of retinal cell homeostasis sustaining [38]. The data collected in our study showed that the level of retinal total ROS and ROS-producer NOX4 was significantly elevated in T1DM rats, while the expression of antioxidant SOD1 was obviously diminished. Moreover, oxidative stress levels and proinflammatory factors, such as IL-1, IL-6, and iNOS, were notably increased in T1DM rats. Recently, studies found that DM was well-recognized as a result of subclinical chronic low-grade inflammation event [39, 40]. Epidemiological studies revealed that levels of inflammatory cytokines, such as IL-6, IL-1*β*, and TNF-*α*, were prominently increased in DM patients [40]. In the case of the dynamic inflammation level alterations in different retinopathy stages, clinical studies showed that serum IL-6 was significantly increased in both the nonproliferative DR and proliferative DR but not in no DR patients with T2DM [26, 41]. Undeniably, higher IL-6 levels are potential risk factors for DR in T2DM. In the present study, diabetic rats with T2DM also showed no statistically significant difference in retinal IL-1 and IL-6 level compared with CON rats. We postulated that 12-week diabetic duration was not the advanced retinopathy stage for T2DM rats.

As we all know, hyperglycemia impairs vascular endothelium resulting in the breakdown of the blood-retinal barrier (BRB) integrity, the enhanced retinal microvascular permeability, and subsequently neovascularization [42–45]. In our animal study, despite the varying increased retinal proangiogenic reaction in both DM, the expression of VEGF-*α* was dramatically lesser in the T2DM group compared with the T1DM group. This relatively slighter angiogenic reaction in the diabetic retina of T2DM was also convincingly verified by the less increasing of HIF-1*α* and VEGFR2, the transcriptional regulator, and receptor of VEGF, respectively. A previous clinical study proved that an increase of serum VEGF level is associated with the severity of diabetic retinopathy in T2DM patients [46]. Particularly, it demonstrated the level of VEGF showed a significant difference between controls and DR, while no significant difference was observed between controls and no DR in patients with clinically T2DM [46]. However, it was reported that the increased level of VEGF was found already in type 1 diabetic children and adolescents without clinical signs of retinopathy or in the early stages of nonproliferative retinopathy [47]. It suggests that VEGF activation occurs in both types of diabetes but increases less radically and slower in T2DM. Our results in the animal study were consistent with these studies. Meantime, it is convinced that the shift in eNOS and iNOS, the endothelial isoform, and inducible isoform of NO synthase, respectively, are implicated in vascular pathologic processes in DR [25, 48]. In line with those studies, we observed a significant increase of the iNOS/eNOS ratio in T1DM retinas. This imbalance was the consequence of oxidative stress injuries and stimulated by proinflammatory cytokines, indicating the presence of radical inflammatory events and vascular endothelial dysfunction in the T1DM retina which somehow explained the poor ERG outcome.

Interestingly, in the present study, mitochondrial ALDH2 was diminished much more in T1DM patients/rats. It was found that ALDH2 was wildly expressed in ONL, inner nuclear layer (INL), and ganglion cell layer (GCL), which featured with an active metabolism. Interestingly, nuclear SIRT1 was also wildly expressed in ONL, INL, and GCL, which was colocated with ALDH2 confirmed by immunofluorescent staining. In addition to that, the distinguished overall mtDNA damage which was extremely sensitive to oxidative stress [33, 36] was observed in the T1DM retina. We speculated that the mitochondrial-nuclear communication between ALDH2 and SIRT1 could alleviate oxidative stress, restore redox homeostasis, and improve the mitochondrion function. To some extent, the present study demonstrated an improved understanding of the ALDH2/SIRT1 interplays, which may elucidate new therapeutic targets for the treatment of DR.

Accordingly, the functional and structural pathological alterations and deleterious oxidative stress reactions have been demonstrated in diabetic rats' retina, especially in T1DM when compared with T2DM. Moreover, the relative lower retinal ALDH2 and SIRT1 expressions, and elevated angiogenic factors were observed in the T1DM retina. Besides, the potential role of ALDH2 in DR and the highly accordant variation tendency of ALDH2 and SIRT1 were once elucidated by our previous work. We supposed that ALDH2/SIRT1 could be related to protecting the retina from ROS damage, alleviating hypoxic ischemic situation and attenuating angiogenic reaction. Thus, it is revealed that ALDH2/SIRT1 may play important roles during physiological and pathological processes of DR.

While the preliminary clinical retrospective observation study demonstrated the potential association between ALDH2/SIRT1 deficiency and DR progression, it is undeniable that the quantity of clinical cases was not sufficient and some essential tests were missed. Therefore, the findings need further thorough study with longer follow-up times for more reliable clinical results to verify the role of ALDH2/SIRT1 in different types of DM during DR development. As for the animal study, the 12-week observation period also seems not long enough and a single detection point was limited. Actually, in the beginning, we were interested in whether T1DM and T2DM showed a different diabetes-related retinal disorder in preclinical DR stage/a certain lasting period. Fortunately, the clinical observations were impressive and some common results were found in animal tests. As for the underlying reason for such difference of retinal ALDH2/SIRT1 and oxidative stress between T1DM and T2DM, further exploration is needed. On the basis of insulin secretion deference, we suspected that the insulin signaling pathway (insulin resistance and reduced glucose utilization), such as phosphoinositide 3-kinase (PI3K)/protein kinase B (AKT) signaling, could be involved in inducing differential protein changes in retinal tissue. Therefore, in the near future, we would like to thoroughly design experiments in both clinical cases and animal models to observe the specific diabetic retinal function and structure changes along with T1DM and T2DM duration and explore the potential role of ALDH2/SIRT1 in the process of diabetic retinal damage.

In summary, we found retinal function and structure injury induced by T1DM was more severe than T2DM in the certain period through clinical and animal study. And we first demonstrated that the slighter retinal disorders in T2DM would be related to the activation of the ALDH2/SIRT1 pathway.

## Figures and Tables

**Table 1:** General characteristics of the included diabetic patients.

Characteristic	T1DM (*n* = 15)	T2DM (*n* = 55)	CON (*n* = 15)	*p* value
Female (%)	6(40.0%)	28(50.9%)	8(53.3%)	
Age (years)	40.3 ± 10.8	52.5 ± 11.5^##^	45.8 ± 15.3	0.002
BMI (kg/m^2^)	24.9 ± 4.0	24.1 ± 3.6	23.5 ± 3.2	ns
Duration of diabetes (years)	11.8 ± 4.5	18.2 ± 6.8^##^		0.001
Systolic blood pressure (mmHg)	124.7 ± 14.3	130.4 ± 16.4	122.2 ± 14.4	ns
Diastolic blood pressure (mmHg)	75.3 ± 7.9	77.3 ± 9.3	76.3 ± 7.4	ns
HbA1c (%)	7.6 ± 1.0^∗∗^	7.5 ± 1.1^∗∗^	5.2 ± 0.6	<0.001
Fasting blood glucose (mmol/L)	8.3 ± 1.1^∗∗^	7.7 ± 1.1^∗∗^	5.2 ± 0.8	<0.001
Therapy components				
Diet only (%)	0	0		
Insulin (%)	15(100%)	51(92.7%)		
Sulfonylureas (%)	0(0%)	8(14.5%)		
*α*-Glucosidase inhibitors (%)	11(73.3%)	31(56.4%)		
Biguanides (%)	9(60.0%)	47 (85.5%)		
Retinal thickness (*μ*m)	279.1 ± 19.8	283.6 ± 18.9	288.9 ± 21.9	ns
ERG test				
Amplitude of max b (*μ*v)	288.5 ± 51.4^∗∗^	337.1 ± 58.9^##^	367.5 ± 47.8	0.001
Amplitude of OPs2 (*μ*v)	86.3 ± 17.8^∗∗^	114.2 ± 20.6^∗∗##^	136.3 ± 19.4	<0.001

The results are described as mean ± standard deviation (SD) or number (%). ns: no significance. ^∗∗^*p* < 0.01: T1DM cohort and T2DM cohort vs. CON cohort; ^##^*p* < 0.01: T2DM cohort vs. T1DM cohort.

**Table 2:** ALDH2 polymorphisms in DM and healthy individuals.

ALDH2 genotype (rs671)	T1DM (*n* = 15)	T2DM (*n* = 55)	CON (*n* = 15)
ALDH2GG (%)	9 (60%)	48 (87.3%)	14 (93.3%)
ALDH2GA (%)	4 (26.7%)	5 (9.1%)	1 (6.7%)
ALDH2AA (%)	2 (13.3%)	2 (3.6%)	0 (0.0%)
ALDH2GA/AA (%)	6 (40.0%)^∗^	7 (12.7%)^#^	1 (6.7%)

The results are described as number (%). ^∗^*p* < 0.05: T1DM cohort and T2DM cohort vs. CON cohort; ^#^*p* < 0.05: T2DM cohort vs. T1DM cohort.

## Data Availability

The data sets used and analyzed in the present study are available from the corresponding authors on reasonable request.

## Abbreviations

4-HNE: 4-Hydroxynonenal
AGEs: Advanced glycation end products
ALDH2: Aldehyde dehydrogenase 2
ALEs: Advanced lipoxidation end-products
BCVA: Best-corrected visual acuity
DM: Diabetes mellitus
DR: Diabetic retinopathy
eNOS: Endothelial nitric acid synthase
ERG: Electroretinograms
GCL: Ganglion cell layer
HIF-1*α*: Hypoxia-inducible factor-1*α*
IL-1: Interleukin-1
IL-6: Interleukin-6
INL: Inner nuclear layer
iNOS: Inducible nitric acid synthase
IS/OS: Inner and outer segment layer
ISCEV: International Society for Clinical Electrophysiology of Vision
MtDNA: Mitochondrial DNA
NAD: Nicotinamide adenine dinucleotide
NOX4: NADPH oxidases 4
OCT: Optical coherence tomography
ONL: Outer nuclear layer
OS: Oxidative stress
SIRT1: Silent information regulator 1
SOD1: Superoxide dismutase1
T1DM: Type 1 diabetes mellitus
T2DM: Type 2 diabetes mellitus
VEGF-*α*: Vascular endothelial growth factor-*α*.
